# GPR142 Controls Tryptophan-Induced Insulin and Incretin Hormone Secretion to Improve Glucose Metabolism

**DOI:** 10.1371/journal.pone.0157298

**Published:** 2016-06-20

**Authors:** Hua V. Lin, Alexander M. Efanov, Xiankang Fang, Lisa S. Beavers, Xuesong Wang, Jingru Wang, Isabel C. Gonzalez Valcarcel, Tianwei Ma

**Affiliations:** 1 Lilly China Research and Development Center (LCRDC), Eli Lilly & Company, Shanghai, China; 2 Lilly Research Laboratories, Lilly Corporate Center, Eli Lilly & Company, Indianapolis, Indiana, United States of America; CRCHUM-Montreal Diabetes Research Center, CANADA

## Abstract

GPR142, a putative amino acid receptor, is expressed in pancreatic islets and the gastrointestinal tract, but the ligand affinity and physiological role of this receptor remain obscure. In this study, we show that in addition to L-Tryptophan, GPR142 signaling is also activated by L-Phenylalanine but not by other naturally occurring amino acids. Furthermore, we show that Tryptophan and a synthetic GPR142 agonist increase insulin and incretin hormones and improve glucose disposal in mice in a GPR142-dependent manner. In contrast, Phenylalanine improves in vivo glucose disposal independently of GPR142. Noteworthy, refeeding-induced elevations in insulin and glucose-dependent insulinotropic polypeptide are blunted in *Gpr142* null mice. In conclusion, these findings demonstrate GPR142 is a Tryptophan receptor critically required for insulin and incretin hormone regulation and suggest GPR142 agonists may be effective therapies that leverage amino acid sensing pathways for the treatment of type 2 diabetes.

## Introduction

Hormones secreted by pancreatic islets and enteroendocrine cells of the gastrointestinal tract play key roles in metabolic homeostasis and diseases including type 2 diabetes (T2D). A clinically validated pathway for T2D therapy is modulation of the incretin hormone glucagon-like peptide-1 (GLP-1), which acts on GLP-1 receptor in pancreatic islets to promote glucose-dependent insulin secretion, thus reducing glucose without causing hypoglycemia. Existing drugs targeting this pathway include Dipeptidyl peptidase-4 inhibitors that stabilize endogenous GLP-1 by inhibiting the key enzyme catalyzing its proteolytic inactivation [1] and injectable GLP-1 receptor agonist peptides that are resistant to DPP-4 cleavage [2]. Furthermore, mechanisms that can enhance the secretion of GLP-1from its endogenous source–enteroendocrine L cells–are also hypothesized to have the potential for robust efficacy in T2D patients without increasing hypoglycemic risk.

Food-derived macronutrients play major roles in regulating postprandial hormonal production from both pancreatic islets and the gut. The effects of various fat derivatives, such as fatty acids and other lipid species, on secretion of insulin and GLP-1 are, at least in part, mediated by several G protein-coupled receptors (GPCRs)[3]. Discovering synthetic agonists for these GPCRs have been the focus of extensive efforts aimed at finding new treatments for T2D. The best known example is TAK-875, a selective agonist for Free Fatty Acid Receptor 1 (FFAR1, GPR40) that is highly expressed in pancreatic islets. TAK-875 activates Gαq signaling downstream of FFAR1 to stimulate glucose-dependent insulin secretion in preclinical models [4], and was shown to improve glycemic control in humans in a phase III study [5], though further development of this molecule was terminated due to safety concerns. In addition, agonists of FFAR1 and other Gαq-coupled GPCRs in enteroendocrine cells can also stimulate the release of gut hormones, including GLP-1, in preclinical models [6]. Therefore, targeting islet/gut Gαq-coupled GPCRs with insulin and GLP-1 secretagogue effects continues to represent an attractive strategy for novel oral T2D treatment.

Dietary polypeptides and amino acids have long been known to stimulate insulin and incretin hormone secretion and regulate postprandial glycemia in animals and in humans [7–10]. Despite reports implicating several cell surface proteins in these effects [11–14], identification of the full repertoire of receptors responsible for sensing of dietary proteins and their derivatives is incomplete. Furthermore, whether these receptors might serve as targets for drug discovery for the treatment of T2D is currently unclear. GPR142 is a Gαq-coupled receptor [15, 16] purported to be selectively activated by L-Tryptophan (L-Trp) [17], yet its biology, including ligand specificity and physiological role in nutrient sensing, remains obscure. *GPR142* mRNA was found to be highly expressed in pancreatic islets in both humans and mice [18, 19], which is consistent with reports of its synthetic agonists stimulating insulin secretion and improving in vivo glucose disposal in mice and primates [20]. The function of GPR142 in other tissues has not been studied but two reports suggested enrichment of *Gpr142* mRNA expression in murine gastric ghrelin cells [21] and intestinal glucose-dependent insulinotropic polypeptide (GIP)-positive K cells [22], respectively. However, the potential connections between the ability of this receptor to sense dietary amino acids, regulate gut hormones secretion, and control glucose metabolism are all unknown.

In this study, we examined GPR142 signaling activity in the presence of various amino acids using a cell-based assay measuring accumulation of D-myo-inositol 1-phosphate (IP-1), a downstream metabolite of D-myo-inositol 1,4,5-triphosphate (IP-3) that is formed upon Gαq activation [23]. We found that the receptor is selectively activated by aromatic amino acids L-Trp and L-Phenylalanine (L-Phe). We further showed that L-Trp and a synthetic GPR142 agonist stimulate the secretion of insulin, the incretin hormones GIP and GLP-1, and improve glucose disposal in mice. The effects on insulin, GIP, and glycemia were absent in *Gpr142* knockout mice. In addition, we showed that GPR142 is a critical modulator of refeeding-induced insulin and GIP release. Interestingly, although L-Phe also reduces glycemia in vivo, its effect remains intact in *Gpr142* knockout mice, suggesting distinct sensing mechanisms for this amino acid. This is the first report to our knowledge demonstrating both the physiological and the pharmacological function of GPR142 in islet and gut hormone secretion and regulation of glucose homeostasis.

## Materials and Methods

### Cellular assays

HEK293 cells obtained from ATCC were used to generate cell lines stably expressing human *GPR142* or mouse *Gpr142* by transfecting cells with pcDNA3.1 expression vectors containing each cDNA. Cells expanded from single clones were maintained in DMEM containing 10% fetal bovine serum (FBS) and 800 μg/ml G418. Untransfected control HEK293 cells were maintained in DMEM containing 10% FBS. Cells were plated in 384 well plates at 5000 cells per well and allowed 18 hours for attachment. After addition of compounds, cells were incubated for 1 hour. IP-1 measurements were performed using an HTRF assay kit (Cisbio) according to manufacturer’s protocol using assay buffer containing 1 × HBSS (Hank’s Balanced Salt Solution, +Ca, +Mg), 0.1% bovine serum albumin (BSA), 50 mM LiCl and 20 mM HEPES (4-(2-hydroxyethyl)-1-piperazineethanesulfonic acid), pH 7.2. The reaction was stopped by addition of IP1-d2 followed by cryptate solution and the plates were incubated at 25°C for 1 hour. Fluorescence was read in an Envision instrument at 665 nm and 620 nm. The ratio of 665 nm/620 nm was calculated and converted to IP-1 levels using a standard curve method. The data was fit to 4-parameter fit logistics to determine EC_50_ values.

#### Experimental animals

db/db mice on C57BL/6 background were obtained from Taconic. *Gpr142* knockout mice (Taconic Farms, 129/B6 background) were backcrossed to C57BL/6 mice (Shanghai Laboratory Animal Center) to achieve >99% C57BL/6 genetic background as determined by SNP genotyping. Animals were maintained in a 12 h light/12 h dark cycle with ambient temperature 22–25°C and allowed ad libitum access to water and food. Diets used were standard rodent chow diet and high-fat diet (D12492, Research Diets). After studies that did not require terminal blood or tissue collection, animals were returned to normal cages and allowed to recover for at least 3 weeks before any other studies that require extensive manipulation or fasting was performed. For studies that required terminal blood or tissue collection, animals were euthanized by CO_2_ inhalation followed by secondary cervical dislocation. All animal procedures were approved by the HD Biosciences (Shanghai), Covance (Shanghai), and Eli Lilly and Company (Indianapolis, IN)’s Institutional Animal Care and Use Committees.

#### Insulin secretion in isolated human pancreatic islets

Human pancreatic islets were purchased from Prodo Labs at the Scharp-Lacy Research Institute (Irvine, CA, www.prodolabs.com) through the Integrated Islet Distribution Program in accordance with internal review board (IRB) ethical guidelines for use of human tissue. Islets were isolated at Prodo Labs from human pancreata from listed cadaver organ donors that were refused for primary human pancreas transplantation or isolated islets transplantion into listed diabetic recipients. Upon receipt, islets were cultured overnight in RPMI-1640 medium (Invitrogen) containing 11 mM glucose and supplemented with 10% (v/v) fetal calf serum and 2 mM glutamine. The next day day islets were incubated for 30 min in EBSS buffer containing 0.1% BSA and 3 mM glucose. Then groups of 3 islets per well were moved to a 48-well plate containing 300 μl EBSS supplemented with 11 mM glucose, 0.1% BSA, and test compounds at indicated concentrations. Islets were cultured for 60 min at 37°C. The incubation was stopped by chilling the samples on ice. The culture media was removed and stored at -20°C until its insulin concentration was measured. Insulin was measured using a custom built immunoassay (Meso Scale Discovery).

### Insulin secretion in isolated murine pancreatic islets

Pancreatic islets were isolated from mice by collagenase digestion and Dextran density gradient separation and were cultured overnight in RPMI-1640 medium containing 11 mM glucose, 10% FBS, and 2 mM glutamine. After overnight recovery, islets were incubated in KRB buffer with 2.8 mM glucose and 0.5% BSA for 45 min, transferred to a 48-well plate with 4 islets per well containing 300 μl/well of compound solutions prepared in appropriate glucose concentrations and 0.1% BSA, and incubated at 37°C for 60 minutes. Secretion was stopped by refrigerating the plates at 4°C for 3 minutes. Supernatant was removed from the wells and assayed for insulin levels using the MA6000 Mouse/Rat Insulin Kit (Meso Scale Discovery).

### RNA extraction and realtime quantitative PCR

Human and mouse pancreatic islets were homogenized in RLT buffer (Qiagen) by vortexing for 1min, and other tissues were homogenized by TissueLyser II (Qiagen) at 30Hz for 2min. Total RNA from human islets and mouse tissues was extracted using RNeasy microkit (Qiagen) with on-column DNase treatment according to the manufacturer’s instructions. Human tissue cDNA panel was purchased from Clontech. 200 ng of RNA was reverse transcribed using the high-capacity cDNA archive kit (Applied Biosystems) or iScript advanced cDNA synthesis kit (Bio-Rad). Realtime PCR was performed using TaqMan Universal Master Mix and the ABI prism 7900 HS sequence detection system (Applied Biosystems). *GPR142* transcripts in mouse islets and mouse and human tissue panels were amplified and detected by the primers and labeled probes listed in S1 Table. *GPR142* transcript in normal vs. diabetic human donor islets were amplified and detected by assay Hs00947114_m1 (Applied Biosystems). The thermal cycling conditions were: 95°C for 10 min and then 40 cycles of 95°C for 15 sec and 60°C for 1 min. Relative expression was calculated by normalization to Rplp0 mRNA by the ΔΔCt method.

### Amino acid challenge, compound treatment, and glucose tolerance test

Mice were fasted overnight and allowed free access to water. The next morning, mice were dosed per os (p.o.) with vehicle (at volume matching compound treatment groups in the same study), L-Trp (20 mL/kg), L-Phe (20 mL/kg), or compound A (10 mL/kg), and glucose solution (2 g/kg, 10 mL/kg) was injected intraperitoneally (i.p.) or gavaged p.o. at indicated time points. Tail blood glucose levels were measured at indicated time points using handheld glucometers. For incretin hormone measurements, blood was collected via cardiac bleeds into EDTA-tubes containing DPP-4 inhibitor (Millipore) and protease inhibitor cocktail (Thermo Fisher). For insulin measurements during GTT, blood was collected from the tail.

### Fasting and refeeding

WT and KO mice were acclimated to single housing for >2 weeks, then randomized to groups 2 days before the study based on non-fasted body weight and blood glucose. After overnight fasting, a group of mice (N = 8 per genotype) was terminated and cardiac blood was collected. The remaining mice were provided pre-weighed normal chow diet and allowed to refeed ad libitum for 30 or 90 minutes (N = 8 per genotype per time point), and cardiac blood was collected for hormone measurements. Diet remaining was weighed and used to calculate food intake. Glucose was measured from tail blood prior to euthanasia.

### Plasma measurements

Immunoassays were used to measure total GIP (Millipore, ELISA), total GLP-1, and insulin (Meso Scale Discovery). Enzymatic assays were used to measure TG, beta-hydroxybutarate (Sigma-Aldrich), and total cholesterol (Thermo Fisher). HDL-cholesterol was determined in the supernatant after removal of LDL/vLDL-cholesterol by a precipitation reagent (Sigma-Aldrich); non-HDL-cholesterol was determined by subtracting HDL from total cholesterol. Free fatty acids were measured by GC-MS.

### Chemicals

Amino acids and amino acid derivatives (≥98% purity) were purchased from Sigma-Aldrich or Acros. Compound A was prepared using a previously described synthetic route [24].

### Statistical analysis

Data were analyzed by Student’s t test, one-way ANOVA with Dunnett’s post-hoc test, or two-way ANOVA with mixed model and Dunnett’s adjustment (with no further multiplicity adjustment between time points). P values less than 0.05 were considered significant.

## Results

### GPR142 is selectively activated by L-Tryptophan and L-Phenylalanine

Beyond Tryptophan, activities of other amino acids on GPR142 have not been described in the literature. To elucidate the biology of GPR142, we first cloned this receptor from primary human and murine pancreatic islets. Noteworthy to mention, two alternative human *GPR142* transcripts are reported to date in Genebank (NCBI Reference Sequences NM_181790.1 and XM_005257305.2) that differ in the first and second exons. In our study in human pancreatic islets, we could only find the transcript XM_005257305.2 that encodes a protein with a shorter N-terminal extracellular domain. The mouse cDNA sequence we have cloned appeared to be identical to the earlier reported XP_006533129 in Refseq.

**Fig 1:**
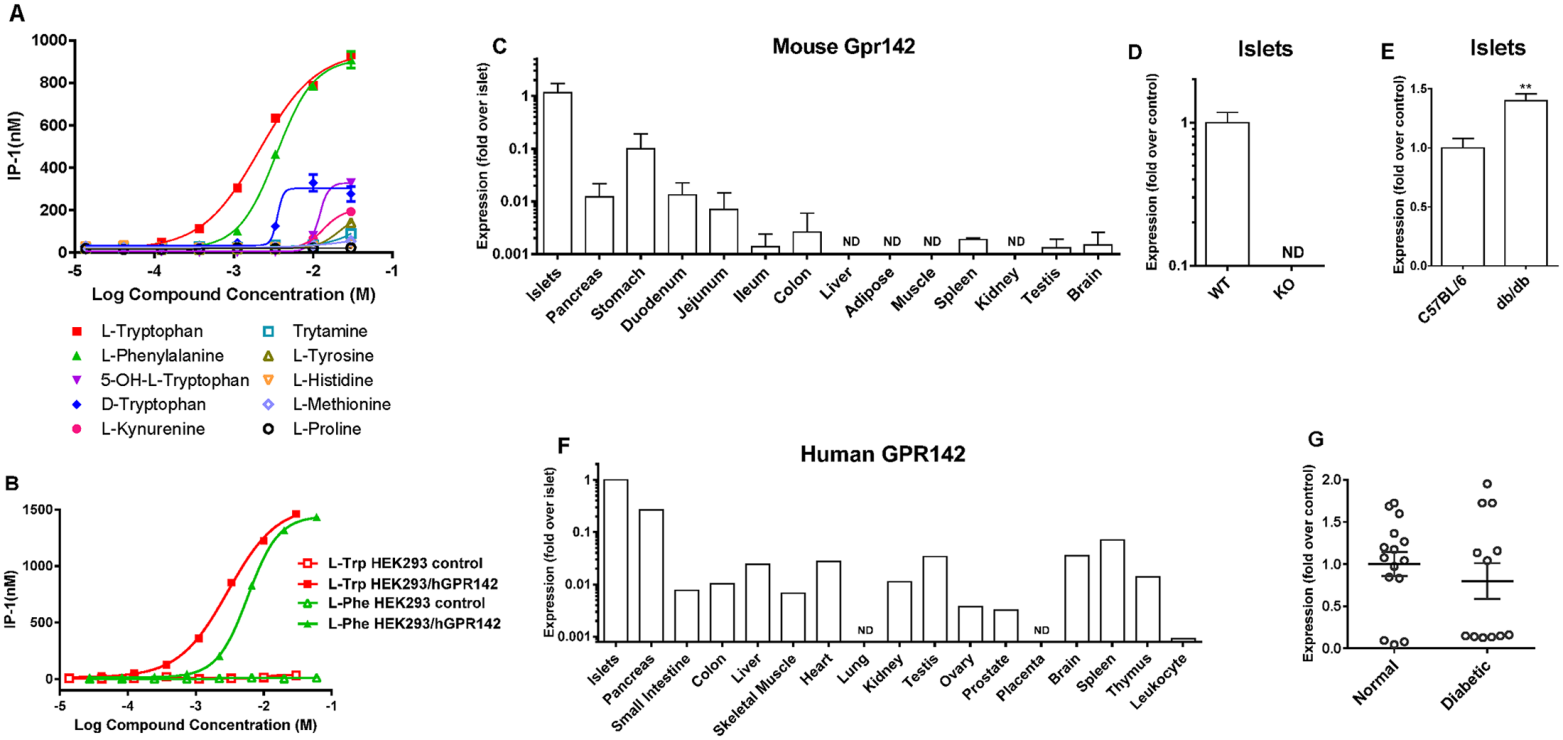
Selective activation of GPR142 signaling by L-Tryptophan and L-Phenylalanine and GPR142 mRNA expression in mouse and human tissues. (A) IP-1 levels in HEK293 cells expressing human GPR142 treated with amino acids and metabolites at varying concentrations. (B) IP-1 levels in HEK293-hGPR142 cells and untransfected control HEK293 cells treated with L-Tryptophan or L-Phenylalanine. Data are mean ± SD of 2 replicate wells. Data representative of 3 independent experiments are shown. (C-E) Mouse *Gpr142* mRNA expression determined by quantitative RT-PCR in (C) tissues of normal C57BL/6 male mice, (D) pancreatic islets isolated from male *Gpr142* KO mice and WT littermate controls, and (E) pancreatic islets isolated from male 8-week-old db/db mice and age-matched C57 control mice. (F, G) Human *GPR142* mRNA expression determined by quantitative RT-PCR in (F) a panel of human tissues and (G) pancreatic islets isolated from healthy non-diabetic or type 2 diabetic donors. Data are normalized against Rplp0 mRNA in each tissue sample, then normalized to the mean value of islet expression (C, F) or control group (D, E, G) set as 1, and expressed as fold expression. N = 2–4 (C-D), N = 5 per group (E), N = 1 pooled biological samples from ≥ 3 donors per tissue type (F), or N = 12–15 donors per group (G). Error bars represent SEM. ND: not detectable. **: p<0.01 control vs. db/db.

We next generated HEK293 cell lines stably expressing either human GPR142 (isoform encoded by XM_005257305.2) or mouse GPR142 (XP_006533129) (HEK-hGPR142 or HEK-mGPR142) and evaluated the effect of amino acids on GPR142 activation by measuring IP-1 levels. We selected HEK293 as the cell background as it does not have detectable endogenous *GPR142* expression and in previous literature has been used to generate GPR142-overexpressing cell lines [17]. As expected, L-Trp stimulated IP-1 accumulation in HEK-hGPR142 and HEK-mGPR142 cells but had no effect in untransfected control HEK293 cells (Fig 1A and 1B). Of interest, L-Phe also has an effect in this assay at concentrations comparable to that of L-Trp. Thus, amino acid-dependent GPR142 activation is not limited to L-Trp only but can also be induced by L-Phe, which is a novel finding. None of the other naturally occurring L-amino acids, metabolites of L-Trp, or D-Trp significantly activated GPR142 signaling at concentrations below 10 mM (Table 1).

**Table 1:** Activities of amino acids, amino acid derivatives, and a synthetic agonist in IP-1 accumulation assays performed in HEK-hGPR142 and HEK-mGPR142 cell lines.

Compounds	HEK293-hGPR142 EC_50_ (Emax)	HEK293-mGPR142 EC_50_ (Emax)
L-Tryptophan	2.1 mM (100%)	0.62 mM (100%)
L-Phenylalanine	3.5 mM (96%)	2.8 mM (92%)
5-OH L-Tryptophan	12 mM (34%)	
D-Tryptophan	3.5 mM (29%)	
L-Kynurenine	12 mM (18%)	
Trytamine	Inactive	
L-Tyrosine	16 mM (14%)	
L-Histidine	Inactive	
L-Methionine	Inactive	
L-Proline	Inactive	
L-Lysine	16 mM (20%)	
L-Arginine	>20 mM (23%)	
L-Aspartic Acid	>20 mM (41%)	ND
L-Asparagine	Inactive	
L-Glutamic Acid	Inactive	
L-Glutamine	Inactive	
L-Leucine	Inactive	
L-Isoleucine	Inactive	
L-Serine	Inactive	
L-Threonine	Inactive	
L-Cysteine	Inactive	
L-Alanine	Inactive	
L-Valine	Inactive	
Glycine	Inactive	
Compound A	7.1 nM (90%)	0.88 nM (90%)

Compounds were tested in concentration response curve ranging from 170 nM to 30 mM except for compound A, which was tested at concentrations ranging from 0.17 nM to 30μM. Relative efficacy (Emax) of each compound was normalized to maximal response of L-Tryptophan (set as 100%). Compounds with maximal response < 10% of L-Tryptophan in the concentration range tested were considered inactive. ND: not determined. Representative data of 3 independent experiments are shown.

### GPR142 is required for L-Trp to stimulate insulin secretion in pancreatic islets

We determined *Gpr142* mRNA expression in various mouse tissues. The tissues with highest *Gpr142* mRNA levels were pancreatic islets, total pancreas, and the proximal gastrointestinal tract—stomach, duodenum, and jejunum (Fig 1C). Analysis of human tissues also showed an enrichment of *GPR142* mRNA in pancreatic islets and total pancreas (Fig 1F), with all other tissues tested showing levels of the mRNA >15-fold lower than that in islets. We also assessed regulation of islet mRNA expression in the diabetic state. *Gpr142*mRNA was slightly elevated in islets isolated from young db/db mice (Fig 1E), whereas no significant difference in mRNA levels was observed between human primary islets from non-diabetic controls and type 2 diabetics (Fig 1G) with high variability in expression level among different donors being noted. It is intriguing that *Gpr142*expression was elevated in islets from db/db mice but not in type 2 diabetic human subjects. Since the db/db mice used in this study were fairly young before the onset of severe diabetes, it is possible they represent an earlier stage in disease progression and have less advanced β cell failure compared to diabetic human donors, which might explain the difference in GPR142 expression observed.

**Fig 2:**
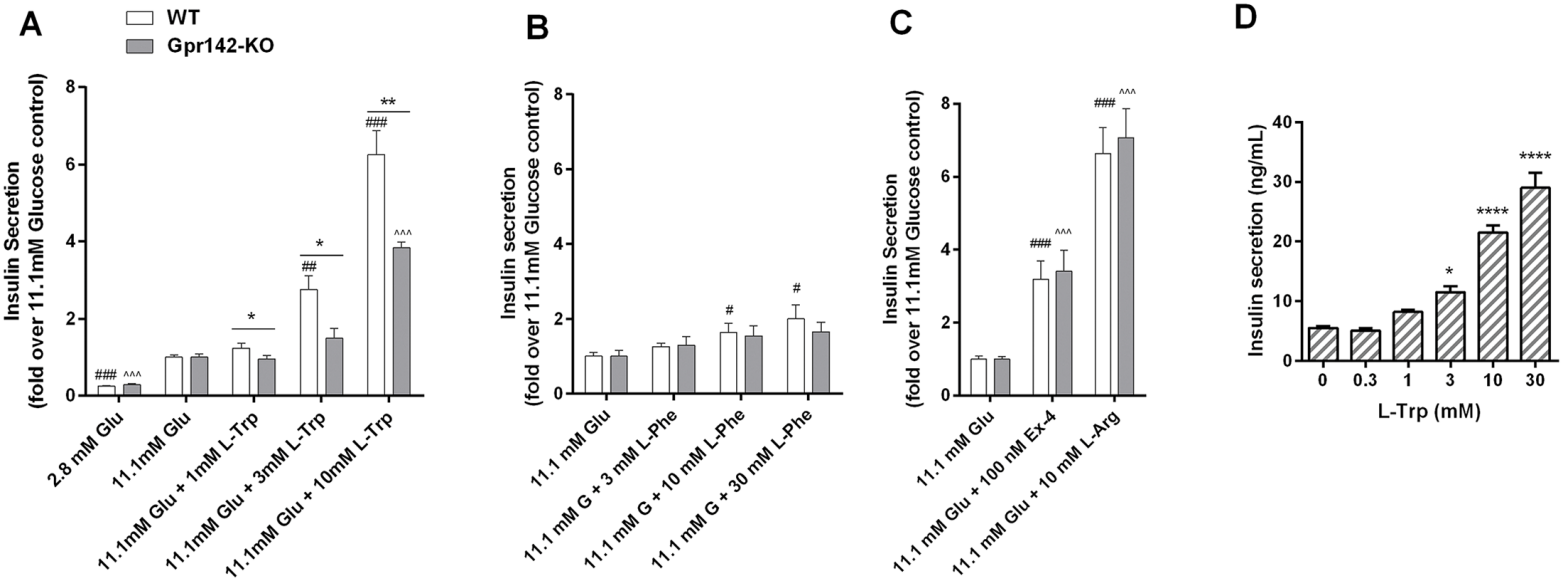
Effects of amino acids on insulin secretion in isolated pancreatic islets. (A-C) Islets isolated from *Gpr142*KO mice and WT littermate controls were incubated in the presence of L-Tryptophan (A), L-Phenylalanine (B), Exendin-4, or L-Arginine (C) for 1 hour, and insulin concentrations in the culture media were measured and expressed as fold change compared to control treatment (11.1 mM glucose). For each experiment, islets were isolated from 8–10 mice, pooled, then plated for treatment. Data are mean ± SEM. N = 5 replicates per treatment group. *,**: p<0.05, 0.01 KO vs. WT; ###: p<0.001 WT islets treatment group vs. 11.1mM glucose control group; ^^,^^^: KO islets p<0.01, 0.001 treatment group vs. 11.1mM glucose control group. (D) Islets isolated from a non-diabetic human donor were incubated at 11.1mM glucose in the presence of varying concentrations of L-Tryptophan, and insulin concentrations in the culture media were measured. Data are mean ± SEM. N = 5 replicates per group. *,****: p<0.05, 0.0001 vs. control (no L-Trp).

Since GPR142 is highly enriched in pancreatic islets, we then studied if GPR142 regulates insulin secretion. We utilized islets from wild type (WT) control mice and *Gpr142*knockout (KO) mice that were confirmed to have receptor expression below the limit of detection by quantitative PCR (Fig 1D). L-Trp dose-dependently stimulated the release of insulin in WT islets in the presence of 11.1 mM glucose (Fig 2A). Importantly, this response was significantly blunted in KO islets (Fig 2A), indicating that GPR142 is a key mediator of the insulin secretagogue activity of L-Trp. L-Trp also robustly stimulated insulin secretion in human primary islets at concentrations similar to those in murine islets (Fig 2D). As of L-Phe, its effect on insulin secretion in WT murine islets was more modest and was not significantly different in islets isolated from *Gpr142*null mice (Fig 2B) altogether making unclear if GPR142 mediates L-Phe’s insulin secretagogue response ex vivo.

Finally, the insulin secretory capacity of WT and KO islets under low glucose or high glucose conditions without exogenous amino acid treatment was not significantly different (WT 0.61±0.06 vs. KO 0.57±0.07 ng/mL/islet/hr at 2.8mM glucose; WT 2.76±0.25 vs. KO 2.40±0.23 ng/mL/islet/hr at 11.1mM glucose). Moreover, islets from WT and KO mice showed similar insulin release upon stimulation with either the GLP-1 receptor agonist Exendin-4 or L-Arginine (Fig 2C), indicative of a specific role of GPR142 in mediating L-Trp regulation of insulin secretion.

### GPR142 is required for L-Trp to improve glucose disposal and stimulate insulin and GIP secretion in vivo

**Fig 3:**
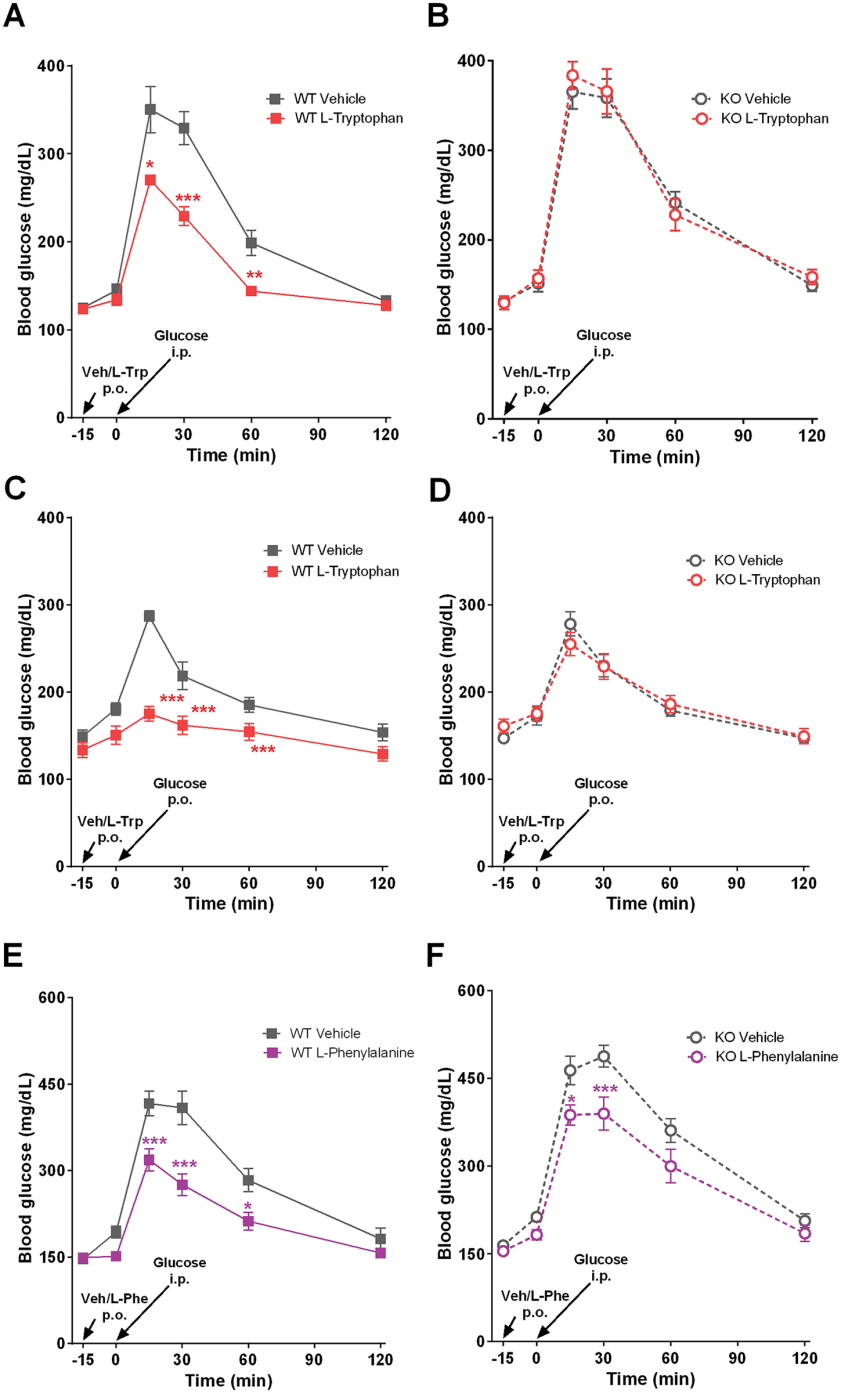
Glucose tolerance tests after L-Tryptophan and L-Phenylalanine oral dosing in *Gpr142*KO mice and WT littermate controls. (A-D) Vehicle (15% HP-β-CD) or L-Trp (500 mg/kg), or (E-F) vehicle or L-Phe (500 mg/kg) was dosed orally at 20 ml/kg to 5hr daytime fasted mice 15 minutes prior to the glucose challenge. Glucose (2 g/kg) was injected i.p. (A-B, E-F) or given by oral gavage (C-D) at indicated time points. Tail blood glucose was monitored for 120 minutes after glucose injection. Animals used were male, 5–8 months of age, and maintained on standard chow diet. Data are mean ± SEM. N = 7 per group. *,**,***: p<0.05, 0.01, 0.001 treatment group vs. vehicle.

We examined the effect of L-Trp and L-Phe on glycemia. Oral dosing of L-Trp suppressed glucose excursion in WT mice during intraperitoneal glucose tolerance test (IPGTT) and oral glucose tolerance test (OGTT) (Fig 3A and 3C). L-Trp had no significant effect on glucose excursion in KO mice (Fig 3B and 3D).Conversely, the effect of L-Phe on glucose excursion during IPGTT was clearly present in both WT and KO mice (Fig 3E and 3F). This finding demonstrates the existence of a novel GPR142-independent pathway that underlies in vivo effect of L-Phe on glucose disposal and warrants additional investigation.

**Fig 4:**
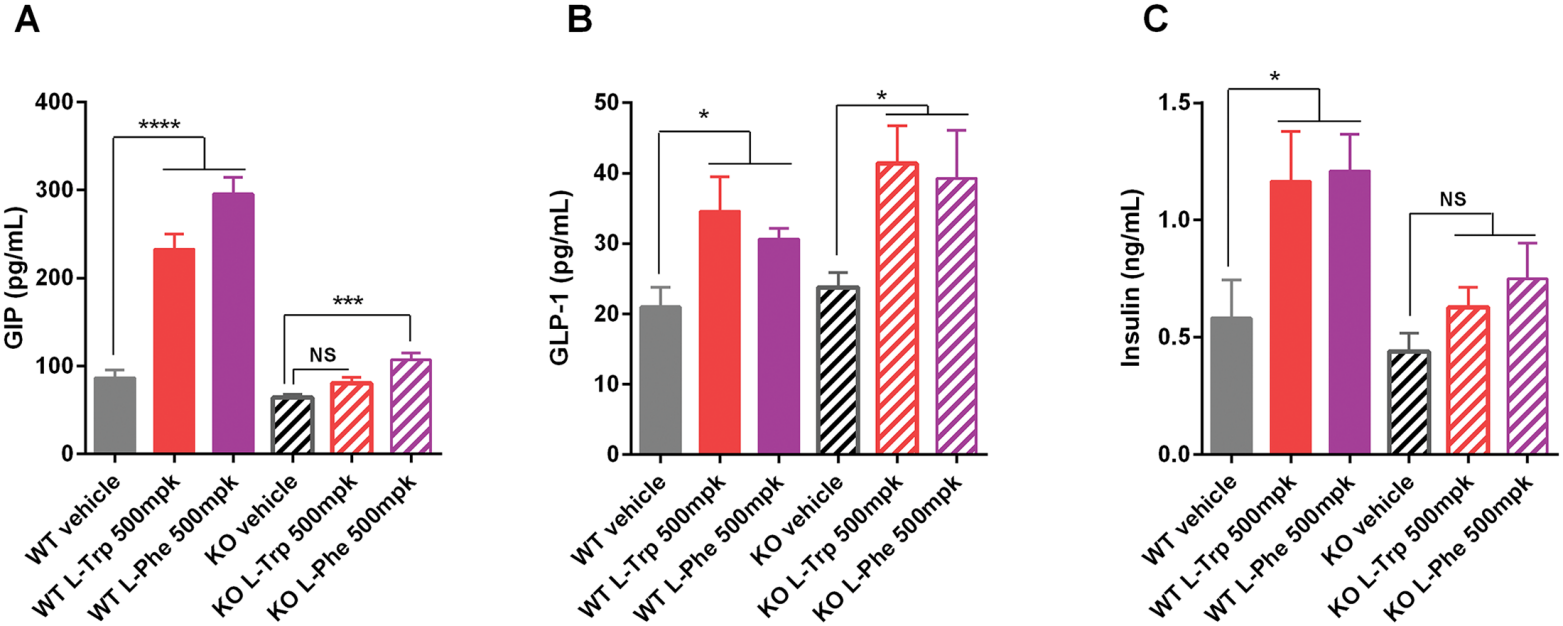
In vivo effects of L-Tryptophan and L-Phenylalanine on plasma hormones in *Gpr142*KO mice and WT littermate controls. Vehicle, L-Trp (500 mg/kg), or L-Phe (500 mg/kg) was dosed orally to overnight fasted male mice and cardiac blood was collected 10 minutes after dosing. Animals were 6–9 months of age, and maintained on standard chow diet. Plasma levels of GIP (A), total GLP-1 (B), and insulin (C) were measured. Data are mean ± SEM. N = 6–7 per group. *,***,****: p<0.05, 0.001, 0.0001 between indicated groups; NS: not significant.

We next examined the effects of L-Trp and L-Phe on insulin and incretin hormones in vivo. Oral dosing of L-Trp rapidly and dose-dependently increased plasma levels of insulin, GIP, and GLP-1, all reaching peak concentrations at 5–10 minutes after dosing (S1A–S1C Fig). We compared the levels of these hormones in WT and KO mice 10 minutes after oral dosing of L-Trp or L-Phe. Noteworthy, no L-Trp-dependent release of GIP and insulin was observed in KO mice (Fig 4A and 4C), while L-Phe induced GIP and insulin release were also blunted in KO mice. This finding indicates that GPR142 is critically required for L-Trp and L-Phe to stimulate these hormones in vivo. Interestingly, both L-Trp and L-Phe increased GLP-1 in KO mice to a similar extent as in WT (Fig 4B). Of importance, no gross abnormality in enteroendocrine K- and L-cell function due to *Gpr142*deficiency was noted in our studies as WT and KO mice showed similar increases in GIP and GLP-1 upon glucose challenge (S2 Fig).

### GPR142 is required for refeeding-induced GIP and insulin secretion in vivo

**Fig 5:**
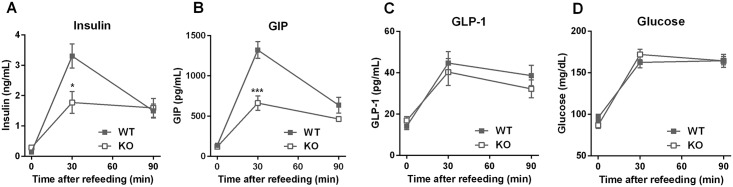
Fasted and refed levels of plasma hormones and blood glucose in *Gpr142*KO mice and WT littermate controls. Plasma levels of insulin (A), GIP (B), total GLP-1 (C), and tail blood glucose (D) were measured after overnight fasting (T = 0), 30-minute refeeding, or 90-minute refeeding. 7-month-old male mice maintained on standard chow diet were used for the study. Data are mean ± SEM. N = 8 per genotype per time point. *,***: p<0.05, 0.001 KO vs. WT.

To understand the role of GPR142 in the regulation of insulin and incretin hormones under physiological feeding conditions, WT and KO mice were subjected to an overnight fast and then allowed ad libitum access to food for 30 or 90 minutes. Baseline body weight and food intake during the refeeding period were not significantly different between WT and KO mice (S2 Table). Upon animals’ refeeding, robust increases were noted in plasma insulin and GIP in WT mice, while these effects were still present but remarkably blunted in KO mice (Fig 5A and 5B). Plasma GLP-1 and blood glucose levels in KO mice after refeeding were not significantly different from WT (Fig 5C and 5D). Thus GPR142 plays a critical role in meal-induced GIP and insulin secretion.

### A synthetic GPR142 agonist stimulates secretion of insulin and incretin hormones and improves glucose tolerance

**Fig 6:**
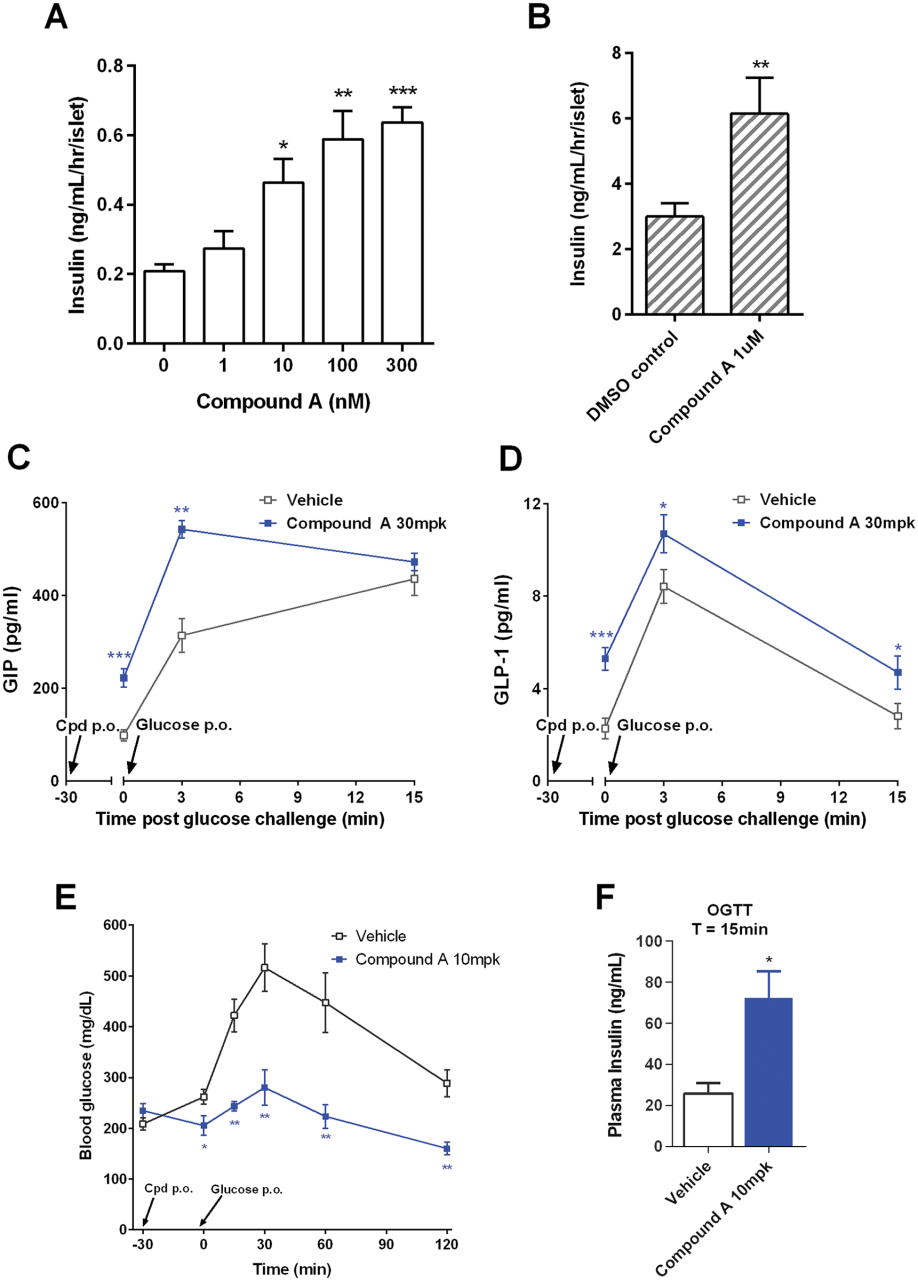
Effects of compound A on insulin secretion, plasma hormones and glycemia during oral glucose tolerance test. (A-B) Pancreatic islets isolated from normal C57 mice (A) or a non-diabetic human donor (B) were incubated in the presence of 11 mM glucose and varying concentrations of compound A for 1 hour. Insulin concentrations in the culture media were measured. Data representative of 2–3 independent experiments are shown. Data are mean ± SEM. N = 5 replicates per group. *,**,***: p<0.05, 0.01, 0.001 vs. control. (C-D) Vehicle (1% w/v HEC, 0.25% v/v Tween80, 0.05% v/v Antifoam in DI water) or compound A (30 mg/kg) was dosed orally to overnight fasted normal male C57 mice. 30 minutes later, glucose (2 g/kg) was orally dosed, and cardiac blood was collected at T = 0 (no glucose dosing), 3, or 15min after glucose challenge. Plasma levels of GIP (C) and total GLP-1 (D) were measured. (E-F) Vehicle or compound A (10 mg/kg) was dosed orally to 5hr daytime fasted diet-induced obese male C57 mice. 30 minutes later, glucose (2 g/kg) was orally dosed. Blood glucose was monitored for the next 120 minutes (E) and plasma insulin at 15 minutes after glucose challenge was measured (F). Data are mean ± SEM. N = 6 per group. *,**,***: p<0.05, 0.01, 0.001 compound A vs. vehicle.

To determine whether effects of L-Trp can be recapitulated by a synthetic GPR142 agonist, we tested compound A [24] (example 49, N-[(3-methylimidazol-4-yl)methyl]-1-[5-methyl-4-(2-thienyl)pyrimidin-2-yl]-5-propyl-pyrazole-4-carboxamide)–a compound that potently stimulates IP-1 downstream of GPR142 signaling in HEK293 cells (Table 1). Treatment with compound A stimulated ex vivo insulin secretion in both murine and human pancreatic islets (Fig 6A and 6B). Oral dosing of compound A in normal C57 mice significantly increased plasma GIP and GLP-1 levels both in the fasted state and after an oral glucose challenge (Fig 6C and 6D). Moreover, compound A treatment led to a robust suppression of glucose excursion during both OGTT and IPGTT in diet-induced mice, a commonly used model of insulin resistance and glucose intolerance, associated with an increase in plasma insulin (Figs 6E and 6F and S3D). The effects of compound A on plasma hormones and glycemia were GPR142-mediated as they were completely absent in KO mice (S3A–S3E Fig). Of note, when dosed prior to an oral mixed meal challenge (Ensure-plus), compound A exerted additional stimulatory effects on meal induced GIP, GLP-1, and insulin levels (S4A–S4C Fig).

## Discussion

L-Trp and L-Phe were both reported to regulate insulin and incretin hormones [25–27]; however the signaling mechanisms mediating these effects have not been delineated and the impact of L-Phe on glycemia has not been characterized. In this study, we demonstrate that activation of the islet-enriched putative amino acid receptor GPR142 is not limited to L-Trp but also observed for L-Phe, which is a novel finding. Using *Gpr142*-deficient mice, we show that L-Trp requires GPR142 for its actions whereas additional as-yet-unidentified pathways contribute to the glucose-lowering effect of L-Phe. This is the first report to our knowledge identifying a GPCR as the Tryptophan receptor responsible for mediating this amino acid’s effects in vivo on gut/islet hormone secretion and glucose homeostasis. The requirement of GPR142 in Tryptophan action appears to be more critical for GIP and insulin secretion than for GLP-1, as L-Trp could still stimulate GLP-1 in *Gpr142*null mice. GLP-1-producing L cells are present at highest density in the lower small intestine and colon, while GIP-producing K cells are restricted to the upper small intestine [28]. Along with the observation that *Gpr142*is expressed at higher levels in the upper small intestine than in distal segments, it is conceivable that an additional L-Trp sensing mechanism may exist in the ileal and colonic L cells to regulate GLP-1 in *Gpr142*-deficient mice.

L-Phe had a weaker effect on ex vivo insulin secretion than L-Trp in murine pancreatic islets. This is consistent with the difference in their potencies on mouse GPR142, since L-Phe is 4 to 5-fold less potent than L-Trp in the HEK293 cell-based IP-1 assay. However, in vivo L-Phe is a robust insulin secretagogue similar to L-Trp, possibly reflecting the aggregate effect of direct activation of GPR142 in pancreatic islets and an indirect effect through incretin hormones on insulin secretion via activating the receptor expressed in the gut. In *Gpr142*-deficient mice, the glucose-lowering effect of L-Phe is still intact, revealing the presence of alternate sensing pathways for this amino acid. A candidate receptor is Calcium-sensing receptor (CaSR), which can be activated by L-amino acids in conjunction with extracellular calcium [29]. Cinacalcet, an allosteric CaSR agonist, was reported to lower glucose in rats [30]. In addition, L-Phe regulation of gut hormones was inhibited by a CaSR antagonist in vitro [31–33]. Worthy of investigation is the physiological role of CaSR as an L-Phe sensor in the regulation of glucose metabolism in vivo, although the pleiotropic effects of CaSR antagonists and severe phenotypes of the whole-body knockout [34] may present technical challenges.

We show that GPR142 is critically required for GIP and insulin secretion stimulated by refeeding. Given the relatively low content of total protein (~20% w/w), Trp and Phe (total ~1% w/w) in normal rodent chow, intuitively it seemed surprising that *Gpr142*deficiency had such a significant impact on GIP and insulin after refeeding (40–50% lower than controls). It is important to note that in the fasting/refeeding experimental setting, animals consumed a very large meal in a short period of time (~35 mg/gram body weight in 30 minutes). Thus the amount of Trp/Phe ingested in the meal (~350 mg/kg body weight) approaches the doses for these amino acids used in pharmacological studies and can be expected to have a major contribution to postprandial hormone secretion. Other macronutrient components of the diet–carbohydrates and fats–presumably can induce incretin and insulin secretion normally in the absence of GPR142, since these hormones in the knockout mice still showed a significant rise after refeeding compared to fasted levels. It is intriguing that postprandial glycemia was not significantly different from control mice despite lower insulin levels in *Gpr142*-deficient mice. Emerging cell type-specific RNAseq data from pancreatic islets suggest GPR142 is not only expressed in β cells but also present in non-β endocrine cells [35] and therefore suggest a possible role in regulation of other islet hormones. Additionally, as GIP can increase glucagon secretion in some contexts [36] while GLP-1 suppresses glucagon [37], GPR142 may also have indirect effects on glucagon secretion via its action on incretin hormones. We did not detect significant differences in glucagon levels in peripheral plasma between *Gpr142*knockout and control mice (data not shown), but cannot rule out a possible role of glucagon in maintaining refed glycemia since peripheral glucagonemia may not reflect its portal concentrations which determines its actions on hepatic glucose output. Additional in vitro islet glucagon secretion studies as well as in vivo measurement of portal glucagon levels may be needed to ascertain a possible role of GPR142 in the regulation of glucagon.

In the absence of an exogenous amino acid challenge, metabolic abnormalities of *Gpr142*-deficient mice are modest, even when subjected to high-fat diet (60 kcal% fat) feeding to induce obesity and metabolic dysfunction (S5 Fig). These include a non-significant trend in increased body weight (S5A Fig), a ~20 mg/dL increase in fasting glycemia (S5B Fig) without any significant change in insulin levels (S5C–S5F Fig), and a 50% increase in plasma non-HDL-cholesterol (S3 Table). The mildness of the metabolic dysfunction in *Gpr142*-deficient mice is not unexpected as multiple pathways likely exist that provide redundancies for optimal protein and amino acid sensing at the whole body level. Furthermore, the phenotype of genetic deficiency for a GPCR does not necessarily predict the efficacy that may be achieved by pharmacological agonists of the same GPCR. Indeed, FFAR1 is an example for which the knockout mice are normoglycemic even when challenged on an insulin resistant background [38, 39], while pharmacological agonists show robust glucose-lowering efficacy.

Finally, we show that a synthetic GPR142 agonist recapitulates the effects of L-Trp on insulin, GIP, and GLP-1, and improves glucose disposal in vivo. This is the first report of GPR142 agonists having a secretagogue effect on incretin hormones, in addition to that on insulin. The insulin secretagogue activities of L-Trp and the synthetic GPR142 agonist were similar in cultured murine islets and human islets, suggesting the glycemic efficacy of the mechanism observed in animals is likely to translate to man. In conclusion, GPR142 agonists may represent a novel therapeutic approach that leverages amino acid sensing pathways in both the gastrointestinal tract and pancreatic islets for the treatment of type 2 diabetes.

## Supporting Information

S1 Fig Plasma hormone levels after L-Tryptophan oral dosing in mice. L-Trp was dosed p.o. at 150 or 500 mg/kg to overnight fasted 8-week-old normal C57 male mice at T = 0. At each indicated time point, cardiac blood was collected from a separate group of mice, and plasma levels of GIP (A), total GLP-1 (B), and insulin (C) were measured. The control group consisted of overnight fasted mice without oral dosing. Data are mean ± SEM. N = 5 per group per time point.
(TIF)

S2 Fig Plasma hormone levels after glucose oral dosing in *Gpr142*KO mice and WT littermate controls. Vehicle or glucose (2 g/kg) was dosed p.o. to overnight fasted KO and WT mice and cardiac blood was collected at 3 minutes after dosing. Plasma levels of GIP (A) and total GLP-1 (B) were measured. 6-month-old female mice maintained on standard chow diet were used for the study. Data are mean ± SEM. N = 6 per group. *,***: p<0.05, 0.001 between indicated groups.
(TIF)

S3 Fig Plasma hormone levels and intraperitoneal glucose tolerance test after oral dosing of compound A in *Gpr142*KO and control mice. (A-C) Vehicle (1% w/v HEC, 0.25% v/v Tween80, 0.05% v/v Antifoam in DI water) or compound A (30 mg/kg) was dosed orally to overnight fasted male KO and WT littermate controls, and cardiac blood was collected 30 minutes after dosing. Plasma levels of GIP (A), total GLP-1 (B), and insulin (C) were measured. >1-year-old male mice maintained on standard chow diet were used for the study. Data are mean ± SEM. N = 7–10 per group. *,**,***,****: p<0.05, 0.01, 0.001, 0.0001 between indicated groups. NS: not significant. (D-E) Vehicle or compound A (30 mg/kg) was dosed orally to overnight fasted male control mice (D) and KO mice (E), 30 minutes later glucose (2 g/kg) was injected i.p., and blood glucose levels were followed for the next 60 minutes. Data are mean ± SEM. 4-month-old male mice maintained on standard chow diet were used for the study. N = 10–15 per group. ***: p<0.001 compound A vs. vehicle at indicated time points.
(TIF)

S4 Fig Plasma hormone levels after oral dosing of compound A and meal. (A-C) Vehicle or compound A (30 mg/kg) was dosed orally to overnight fasted normal male C57 mice. 30 minutes later, water or Ensure-plus (10 mL/kg) was dosed orally, and cardiac blood was collected 5 minutes afterwards. Plasma levels of GIP (A), total GLP-1 (B), and insulin (C) were measured. Data are mean ± SEM. N = 5 per group. *,**,***,****: p<0.05, 0.01, 0.001, 0.0001 vs. vehicle + water control group. #,##: p<0.05, 0.01 between indicated groups.
(TIF)

S5 Fig Metabolic phenotypes of WT and *Gpr142*KO mice maintained on high-fat diet (HFD). Mice on C57BL/6 genetic background were fed HFD starting from 6 weeks of age, and various metabolic parameters were measured. Body weight (A) was measured for 22 weeks. Blood glucose (B) and plasma insulin levels (C) during an OGTT, food intake (D), ad libitum fed blood glucose (E) and plasma insulin levels (F) in male *Gpr142*KO and WT littermates were measured after 11–15 weeks of HFD feeding. Data are mean ± SEM. N = 12 per group. *: p<0.05 WT vs. KO.
(TIF)

S1 Table Sequences of primers and probes used for GPR142 Taqman analysis. (DOCX)

S2 Table Body weight and refeeding food intake of WT and Gpr142 KO mice maintained on standard chow diet. (DOCX)

S3 Table Metabolic characteristics of WT and Gpr142 KO mice on high-fat diet. (DOCX)
