# Supplementary figures and images for: GPR142 Controls Tryptophan-Induced Insulin and Incretin Hormone Secretion to Improve Glucose Metabolism
Source: PLoS One. 2016 Jun 20;11(6):e0157298. doi: 10.1371/journal.pone.0157298 (PMC4920590; doi:10.1371/journal.pone.0157298)

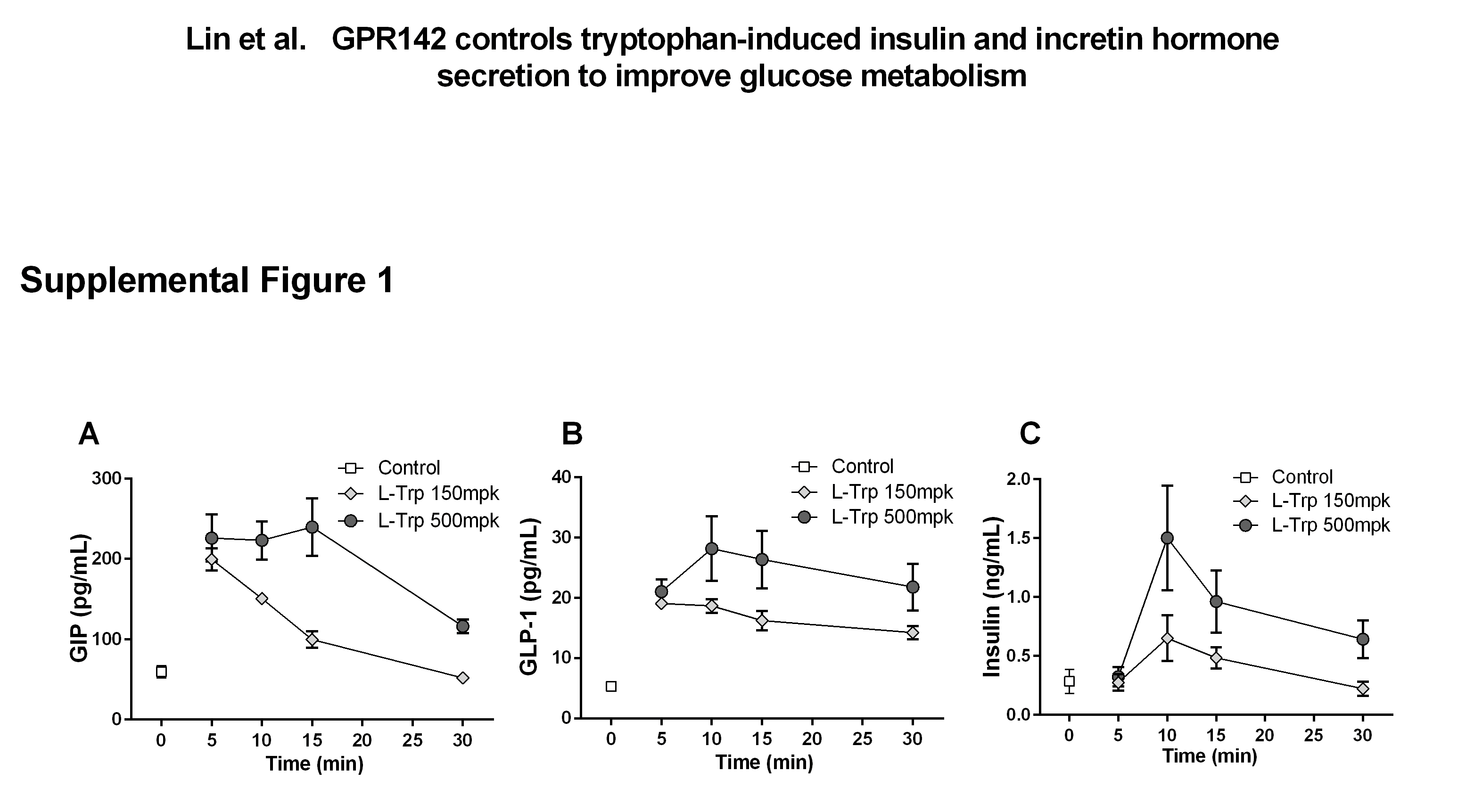

Supplement: S1 Fig — L-Trp was dosed p.o. at 150 or 500 mg/kg to overnight fasted 8-week-old normal C57 male mice at T = 0. At each indicated time point, cardiac blood was collected from a separate group of mice, and plasma levels of GIP (A), total GLP-1 (B), and insulin (C) were measured. The control group consisted of overnight fasted mice without oral dosing. Data are mean ± SEM. N = 5 per group per time point. (TIF) [file pone.0157298.s001.tif]

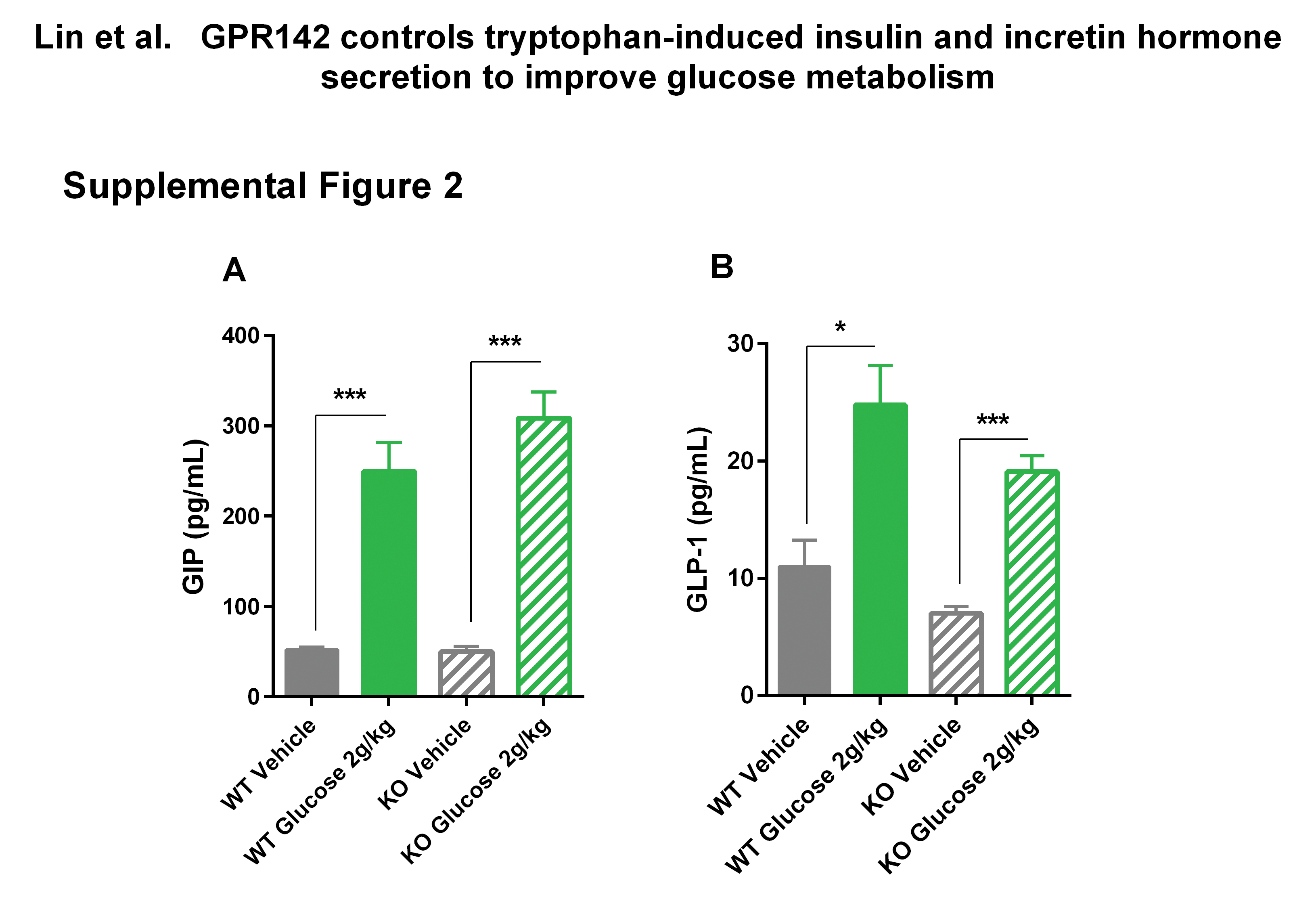

Supplement: S2 Fig — Vehicle or glucose (2 g/kg) was dosed p.o. to overnight fasted KO and WT mice and cardiac blood was collected at 3 minutes after dosing. Plasma levels of GIP (A) and total GLP-1 (B) were measured. 6-month-old female mice maintained on standard chow diet were used for the study. Data are mean ± SEM. N = 6 per group. *,***: p<0.05, 0.001 between indicated groups. (TIF) [file pone.0157298.s002.tif]

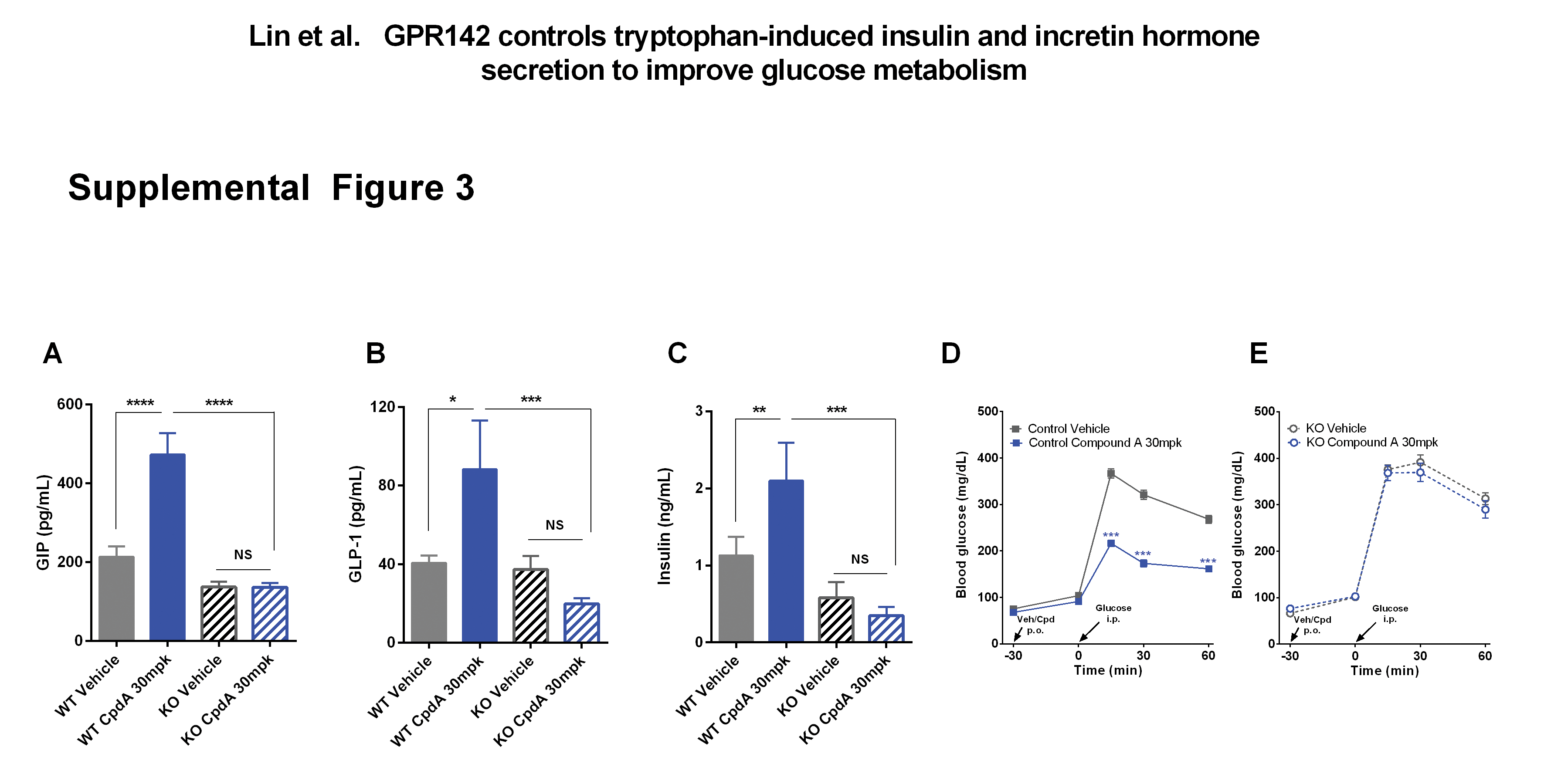

Supplement: S3 Fig — (A-C) Vehicle (1% w/v HEC, 0.25% v/v Tween80, 0.05% v/v Antifoam in DI water) or compound A (30 mg/kg) was dosed orally to overnight fasted male KO and WT littermate controls, and cardiac blood was collected 30 minutes after dosing. Plasma levels of GIP (A), total GLP-1 (B), and insulin (C) were measured. >1-year-old male mice maintained on standard chow diet were used for the study. Data are mean ± SEM. N = 7–10 per group. *,**,***,****: p<0.05, 0.01, 0.001, 0.0001 between indicated groups. NS: not significant. (D-E) Vehicle or compound A (30 mg/kg) was dosed orally to overnight fasted male control mice (D) and KO mice (E), 30 minutes later glucose (2 g/kg) was injected i.p., and blood glucose levels were followed for the next 60 minutes. Data are mean ± SEM. 4-month-old male mice maintained on standard chow diet were used for the study. N = 10–15 per group. ***: p<0.001 compound A vs. vehicle at indicated time points. (TIF) [file pone.0157298.s003.tif]

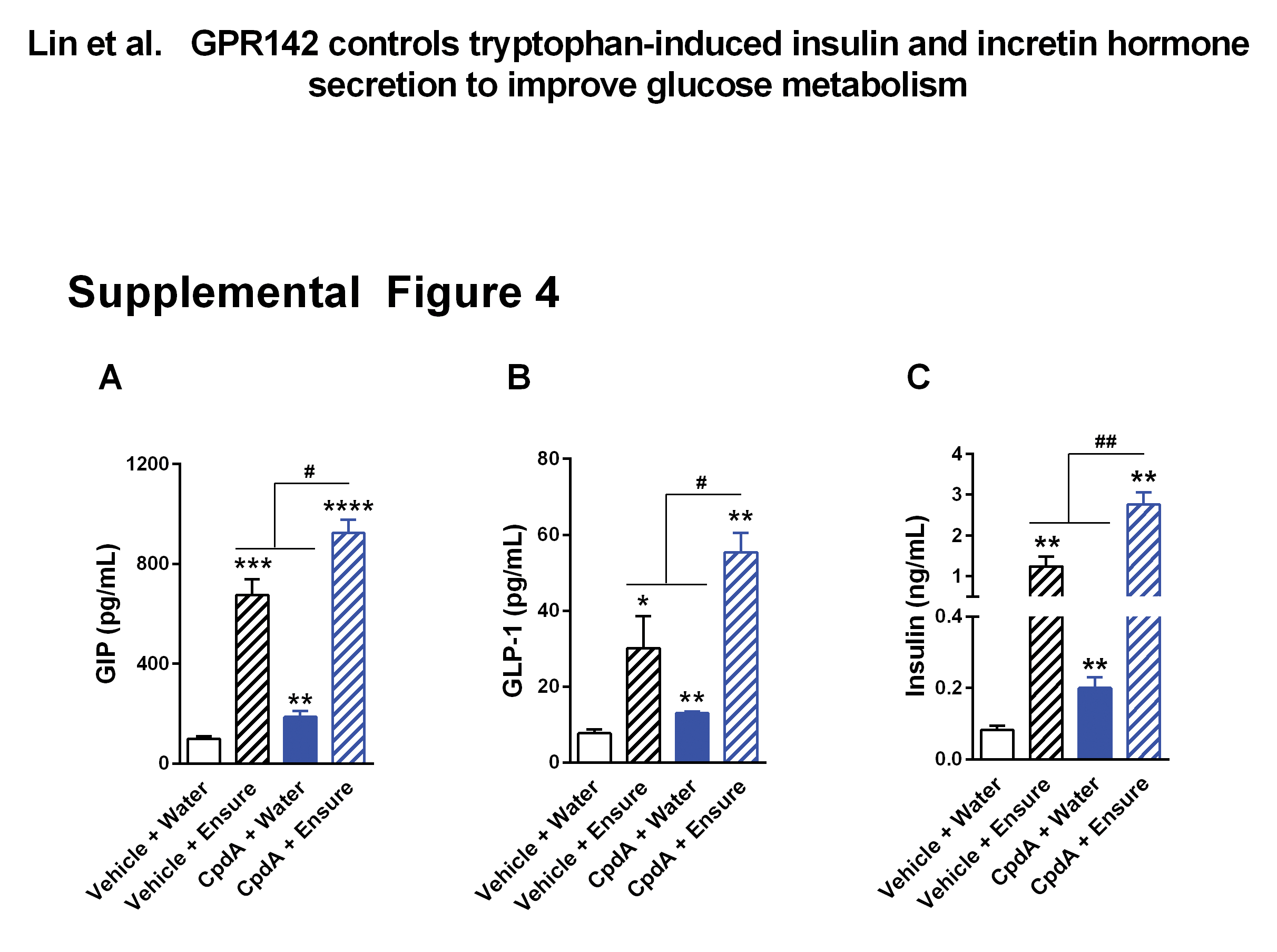

Supplement: S4 Fig — (A-C) Vehicle or compound A (30 mg/kg) was dosed orally to overnight fasted normal male C57 mice. 30 minutes later, water or Ensure-plus (10 mL/kg) was dosed orally, and cardiac blood was collected 5 minutes afterwards. Plasma levels of GIP (A), total GLP-1 (B), and insulin (C) were measured. Data are mean ± SEM. N = 5 per group. *,**,***,****: p<0.05, 0.01, 0.001, 0.0001 vs. vehicle + water control group. #,##: p<0.05, 0.01 between indicated groups. (TIF) [file pone.0157298.s004.tif]

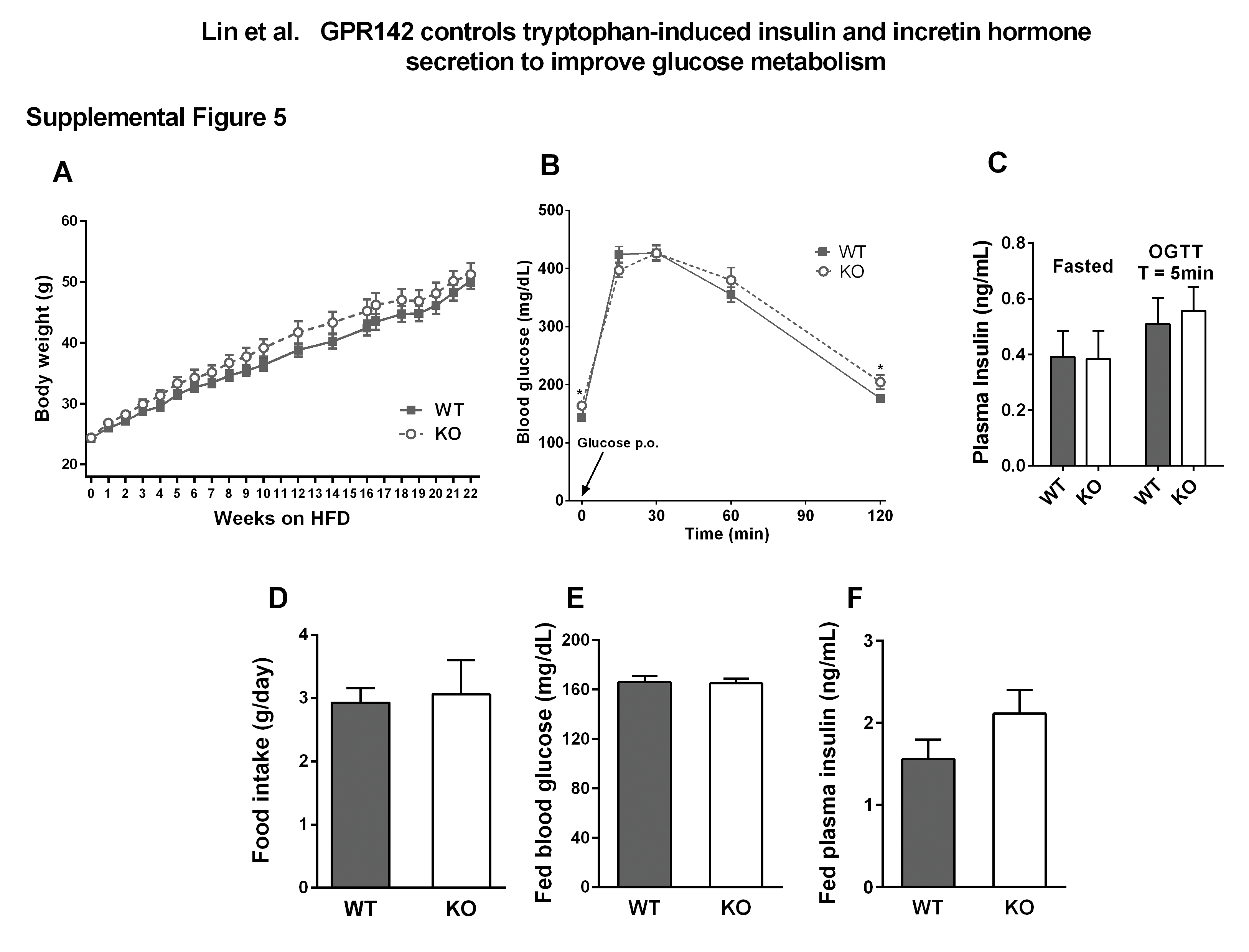

Supplement: S5 Fig — Mice on C57BL/6 genetic background were fed HFD starting from 6 weeks of age, and various metabolic parameters were measured. Body weight (A) was measured for 22 weeks. Blood glucose (B) and plasma insulin levels (C) during an OGTT, food intake (D), ad libitum fed blood glucose (E) and plasma insulin levels (F) in male Gpr142KO and WT littermates were measured after 11–15 weeks of HFD feeding. Data are mean ± SEM. N = 12 per group. *: p<0.05 WT vs. KO. (TIF) [file pone.0157298.s005.tif]
