# Supplementary material for: GPR142 Controls Tryptophan-Induced Insulin and Incretin Hormone Secretion to Improve Glucose Metabolism
Source: PLoS One. 2016 Jun 20;11(6):e0157298. doi: 10.1371/journal.pone.0157298 (PMC4920590; doi:10.1371/journal.pone.0157298)
Supplement: S1 Table — (DOCX) [file pone.0157298.s006.docx]

**Supplementary Table 1**. Sequences of primers and probes used for GPR142 Taqman analysis.

| Species | Position | Primer 1 | Primer 2 | Probe |
| --- | --- | --- | --- | --- |
| Mouse | Exon2-3 | GGACCCAGGACCCAGAACTATG | GCCATCCACACCATCGTATC | CCTGCTGCCTACAGTCAATGGTAGTA |
| Mouse | Exon3-4 | CCCAAGATACGATGGTGTGGAT | CCGTGAGTGCCAGGAGATAG | CAGTGGCACTGGCCCGTCTTG |
| Human | Exon3-4 | GGGCTGAGCCAGGAGTTTG | ACTGCGGTCAGGAGGCTGA | AAAGCCACTGGCCAGAGATCGC |
