# Supplementary material for: GPR142 Controls Tryptophan-Induced Insulin and Incretin Hormone Secretion to Improve Glucose Metabolism
Source: PLoS One. 2016 Jun 20;11(6):e0157298. doi: 10.1371/journal.pone.0157298 (PMC4920590; doi:10.1371/journal.pone.0157298)
Supplement: S2 Table — (DOCX) [file pone.0157298.s007.docx]

**Supplementary Table 2**. Body weight and refeeding food intake of WT and Gpr142 KO mice maintained on standard chow diet.

| Genotype | WT | Gpr142 KO | P value |
| --- | --- | --- | --- |
| Baseline body weight (g) | 33.7 ± 0.6 | 33.2 ± 0.5 | 0.49 |
| 30min refeeding food intake  (mg/g baseline body weight) | 33.3 ± 1.9 | 30.3 ± 1.1 | 0.15 |
| 90min refeeding food intake  (mg/g baseline body weight) | 43.7 ± 2.6 | 36.6 ± 3.3 | 0.10 |

Baseline body weight before overnight fasting of male WT and Gpr142 KO mice maintained on standard chow diet. After fasting, animals were allowed ad libitum access to standard chow diet, and refeeding food intake during 30 or 90 minutes was measured. Data are mean ± SEM.
