# Supplementary material for: GPR142 Controls Tryptophan-Induced Insulin and Incretin Hormone Secretion to Improve Glucose Metabolism
Source: PLoS One. 2016 Jun 20;11(6):e0157298. doi: 10.1371/journal.pone.0157298 (PMC4920590; doi:10.1371/journal.pone.0157298)
Supplement: S3 Table — (DOCX) [file pone.0157298.s008.docx]

**Supplementary Table 3**. Metabolic characteristics of WT and Gpr142 KO mice on high-fat diet.

| Genotype | WT | Gpr142 KO |
| --- | --- | --- |
| Triglycerides (mg/dL) | 82.3 ± 5.9 | 79.7 ± 3.6 |
| β-hydroxybutyrate (mM) | 0.79 ± 0.04 | 0.67 ± 0.05 |
| HDL-cholesterol (mg/dL) | 169 ± 13 | 171 ± 11 |
| non-HDL-cholesterol (mg/dL) | 41.9 ± 4.1 | 62.0 ± 6.0* |
| NEFA C16:0, C18:0, C18:1, C18:2 (arbitrary unit) | 1.14±0.03, 0.51±0.02, 0.20±0.01, 0.12±0.01 | 1.17±0.04, 0.53±0.02, 0.20±0.01, 0.13±0.01 |
| Liver Weight (g) | 1.79 ± 0.19 | 1.61 ± 0.14 |
| Liver TG (mg/g tissue) | 160 ± 22 | 159 ± 22 |
| Pancreas Weight (g) | 0.28 ± 0.01 | 0.28 ± 0.01 |
| Inguinal fat pad weight (g) | 2.73 ± 0.13 | 3.25 ± 0.30 |
| Epididymal fat pad weight (g) | 2.63 ± 0.20 | 2.65 ± 0.16 |

Plasma parameters were measured in male mice on HFD for 18 weeks after overnight fasting. Levels of non-esterified fatty acids (NEFA) are measured by GC-MS and expressed as relative abundance. Tissue weights and liver TG content were measured in mice on HFD for 22 weeks after overnight fasting. Data are mean ± SEM. N=12 male mice per genotype. *: p<0.05 WT vs. KO.
